# Selective Reinforcement Optimization for Composite Laminates

**DOI:** 10.3390/ma19020305

**Published:** 2026-01-12

**Authors:** Artem Balashov, Anna Burduk, Michał Krzysztoporski, Piotr Kotowski

**Affiliations:** Faculty of Mechanical Engineering, Politechnika Wroclawska, ul. I. Łukasiewicza 5, 50-370 Wroclaw, Poland

**Keywords:** selective reinforcement optimization, composite material, DBSCAN, Tsai–Wu criterion, structural optimization, additive manufacturing

## Abstract

Composite laminates designed for additive manufacturing require efficient material distribution to minimize weight while maintaining structural integrity. Traditional topology optimization methods, however, produce continuous density fields incompatible with layer-based fabrication. This work presents Selective Reinforcement Optimization (SRO), a stress-driven methodology that converts uniformly loaded laminate layers into localized reinforcement regions, or “patches”, at critical stress concentrations. The approach employs layer-wise statistical analysis of Tsai–Wu failure indices to identify high-variance layers; applies DBSCAN clustering to extract spatially coherent stress regions while rejecting artificial concentrators; and generates CAD-compatible and manufacturing-ready boundary geometries through a custom concave hull algorithm. The method operates iteratively in dual modes: lightweighting progressively removes full layers and replaces them with localized regions when the structure is safe, while strengthening adds reinforcement without layer removal when failure criteria are approached. Case studies demonstrate weight reductions of 10–30% while maintaining failure indices below unity, with typical convergence achieved within 100 iterations. Unlike classical topology optimization, which requires extensive post-processing, SRO directly outputs discrete patch geometries compatible with composite additive manufacturing, offering a computationally efficient and production-oriented framework for the automated design of layered composite structures.

## 1. Introduction

Composite laminates have become foundational in modern engineering due to their high stiffness-to-weight ratio, tailorability, and compatibility with advanced manufacturing processes. Their adoption across aerospace, renewable energy, automotive, and civil structures has accelerated the demand for design methodologies that can efficiently distribute material, reduce weight, and maintain structural safety under complex loading scenarios. As laminates become increasingly used in additive and automated manufacturing workflows, the limitations of traditional design paradigms—particularly those derived from isotropic materials—become more obvious. The central challenge lies in the spatially heterogeneous nature of laminate stresses, the discrete stacking architecture, and the manufacturing constraints intrinsic to layer-by-layer construction [1,2].

Finite element analysis (FEA) remains the primary computational tool for evaluating composite laminates, enabling engineers to simulate layer-wise stress states, interlaminar failure modes, and failure indices based on composite-specific criteria such as Tsai–Wu [3]. While these simulations provide detailed insight into structural performance, extracting actionable design guidance from stress fields remains a nontrivial task. Classical optimisation techniques—sizing, shape, and topology optimisation—have all been used to improve composite structures. However, their compatibility with laminated, fibre-oriented materials is often limited. Density-based topology optimisation, for example, generates greyscale distributions that are not directly manufacturable in composite layups, as material cannot be deposited in continuously varying densities [4]. Similarly, shape optimisation methods commonly assume smooth, continuum-based boundaries whose geometric adjustability does not map naturally to discrete reinforcement patches in laminate design.

Recent developments in composite-specific optimisation methods demonstrate growing interest in bridging this gap. For example, concurrent topology and fibre-orientation optimisation frameworks aim to adapt both material distribution and local fibre directions simultaneously [5]. These methods leverage advanced numerical regularisation to maintain manufacturable, continuous fibre–field descriptions. Yet, they still face two practical limitations: (1) they fundamentally assume continuous fields, whereas composite additive manufacturing deals with discrete layers; and (2) their outputs typically require post-processing before they can be translated into production-ready geometries. Other strands of research focus on reliability analysis, probabilistic lamina evaluation, and automated modelling workflows [6,7], highlighting the importance of statistical reasoning and automation in composite design. These developments demonstrate that the field increasingly recognises the need for optimisation approaches that are directly compatible with digital manufacturing pipelines.

At the same time, a parallel evolution in intelligent design practices shows growing interest in data-driven decision systems embedded into manufacturing workflows [7]. Python-driven automation frameworks for Abaqus and similar FEA tools have enabled engineers to script large optimisation loops, manipulate laminates parametrically, and extract detailed stress information for on-the-fly decision-making [8]. These capabilities open the door for hybrid optimisation strategies that combine FEA, statistical inference, and computational geometry—strategies that align closely with industrial design constraints such as cut-and-place patching, defect-tolerant reinforcement, and iterative build–test cycles.

Despite these advancements, a noticeable methodological gap remains. Existing optimisation methods rarely exploit one of the central characteristics of laminated composites: the fact that load distribution across plies is not uniform, and that some layers may experience large stress gradients while others remain nearly uniform. A laminate may contain layers where stresses are highly concentrated in small regions, while other layers carry load more uniformly. Yet classical tools treat laminate plies as components whose thickness or fibre angle must be globally modified, instead of locally redistributed. In practice, manufacturers often apply reinforcement patches—small cut-out regions of additional plies—only in locations where stress concentrations or damage are expected. However, no established optimisation method exists that systematically automates such patch placement using mathematically robust, FEA-driven stress analysis.

The key limitation is the absence of an algorithmic framework capable of converting layer-wise stress heterogeneity into geometrically well-defined reinforcement patches. Classical methods are either too global (topology optimisation), too continuous (fibre-orientation optimisation), or too coarse (rule-based ply-drop heuristics). Meanwhile, composite additive manufacturing and automated lay-up systems increasingly rely on geometrically explicit commands—closed curves, toolpaths, and cut patterns—rather than abstract field descriptions. Therefore, a design methodology that outputs discrete, manufacturable reinforcement geometries directly from stress fields is increasingly needed.

To meet this need, the present work introduces Selective Reinforcement Optimization (SRO), a stress-driven design methodology developed by the author. SRO uses a fundamentally different optimisation perspective: instead of increasing or decreasing thickness across the entire laminate, it identifies stress concentrations within layers and transforms under-utilised material into localized reinforcement patches. This approach redistributes material rather than adding it, adhering more closely to composite manufacturing constraints. Its workflow begins with ply-wise FEA to compute the Tsai–Wu failure index field for each layer [3]. From this, the algorithm evaluates the statistical variability of each layer’s stress distribution using measures such as the coefficient of variation (CV). High-variance plies—those exhibiting sharp localised peaks—are considered candidates for reinforcement redistribution, while low-variance plies are considered structurally uniform.

The extracted stress fields are then filtered using percentile-based statistical thresholds, an approach grounded in established principles of robust statistical modelling [9]. Spatial clustering is performed using DBSCAN [10], which is well-suited for identifying arbitrary-shaped stress regions while rejecting artificial stress concentrators caused by mesh irregularities or poorly constrained boundary conditions. To transform clusters into manufacturable geometries, SRO applies algorithmic boundary extraction, favouring α-shapes [11] for precise concave boundary resolution while also acknowledging that faster custom boundary methods may be acceptable for manufacturing, where the precision of patch placement is naturally constrained.

This methodology provides an optimisation pathway that is inherently discrete, geometry-oriented, and directly compatible with automated composite fabrication steps such as cut-and-place patching, tape layup, or robotic fibre steering. Moreover, because it functions iteratively, it can operate in dual modes: lightweighting (removing full plies and converting them into patches when the structure remains safe) or strengthening (adding patches without removing plies when the laminate approaches failure).

In this context, the development of SRO can be seen as a response to the increasing convergence of FEA automation, statistical modelling, composite manufacturing constraints, and computational geometry. The purpose of this work is to formally present the SRO methodology, situate it within the broader landscape of composite optimisation research, and demonstrate its capability to generate manufacturing-ready reinforcement geometries directly from stress fields. The results show that SRO can reduce weight while ensuring structural safety, offering a practical alternative to continuous topology optimisation methods for laminate-based structures.

Recent numerical investigations of laminated thin plates have emphasised the importance of modal and stress-mode analyses for understanding how layup and local stress components dictate modal participation and stress concentration patterns [12]. These studies demonstrate that ply-level modal and stress localisation effects can be exploited by targeted geometric or material interventions, reinforcing the premise that layer-wise information contains actionable design cues that can be used to generate discrete reinforcement geometries rather than global, continuous fields.

From an engineering perspective, related work on the performance and robustness of composite and composite-like structural systems under long-term and multi-hazard loading highlights the practical significance of design methods that explicitly account for direction-dependent and case-specific demands [13,14,15]. Additionally, recent optimisation studies focused on wind turbine blades and hybrid fibre-reinforced composites demonstrate the value of combining structural simulation with tailored reinforcement strategies to achieve both strength and mass objectives [16,17]. These contributions motivate the present focus on a discrete, manufacturable reinforcement paradigm that can respond to directional and case-dependent stress features identified at the ply level.

## 2. Materials and Methods

### 2.1. Mathematical Formulation

Let the composite laminate consist of *L* plies (laminae), indexed j=1,…,L, each with material properties $M_{j}$ and fiber orientation $\theta_{j}$. Let N denote the set of finite element nodes. For each layer *j*, we define the nodal Tsai–Wu failure index field from FEA as

$$F^{\left(j\right)}=\left\{F_{i}^{\left(j\right)}\;:\;i\in N\right\},$$

where $F_{i}^{\left(j\right)}$ is the Tsai–Wu failure criterion [18] value at node *i* in layer *j*. The Tsai–Wu criterion is defined as

$$F_{i}^{\left(j\right)}=f_{1}\sigma_{11}+f_{2}\sigma_{22}+f_{11}\sigma_{11}^{2}+f_{22}\sigma_{22}^{2}+f_{66}\tau_{12}^{2}+2f_{12}\sigma_{11}\sigma_{22},$$

where the coefficients $f_{•}$ are material-dependent and determined from lamina strength properties. A value $F_{i}^{\left(j\right)}\geq 1$ indicates failure.

#### 2.1.1. Layer-Wise Statistical Characterisation

The mean and standard deviation of the failure field in layer *j* are

(1) $$\begin{array}{cc} \mu_{j} & =\frac{1}{\left|N\right|}\sum_{i\in N}F_{i}^{\left(j\right)}, \end{array}$$

(2) $$\begin{array}{cc} \sigma_{j} & =\sqrt{\frac{1}{\left|N\right|}\sum_{i\in N}{\left(F_{i}^{\left(j\right)}-\mu_{j}\right)}^{2}}. \end{array}$$

The coefficient of variation (relative dispersion) quantifies stress non-uniformity within a layer as follows:

(3) $${\mathrm{CV}}_{j}=\frac{\sigma_{j}}{\mu_{j}}.$$

A high ${\mathrm{CV}}_{j}$ indicates that stress is concentrated in specific regions of layer *j*, making it an optimal candidate for selective reinforcement. The target layer for optimization is

(4) $$j^{*}=\arg\max_{j=1,\ldots ,L}{\mathrm{CV}}_{j},$$

subject to the constraint $\mu_{j}>\mu_{\min}$ to avoid selecting entirely unloaded layers.

#### 2.1.2. Statistical Node Filtering

To isolate statistically significant high-failure regions while rejecting outliers and artificial stress concentrators, we employ a percentile-based threshold.

Let $P_{1-\beta }\left(F^{\left(j^{*}\right)}\right)$ denote the (1−β) percentile of the Tsai–Wu distribution in layer $j^{*}$. The set of critical nodes is

(5) $$H^{\left(j^{*}\right)}=\left\{i\in N:F_{i}^{\left(j^{*}\right)}>P_{1-\beta }\left(F^{\left(j^{*}\right)}\right)\right\},$$

where β∈(0,1) controls the patch size.

#### 2.1.3. Adaptive Threshold Selection

The percentile parameter is updated each iteration based on structural performance as follows:

(6) $$\beta^{\left(k+1\right)}=\beta^{\left(k\right)}\cdot \left\{\begin{array}{cc} \gamma_{\mathrm{up}} & \mathrm{if}\;F_{\max}^{\left(k\right)}>F_{\mathrm{target}},\\ \gamma_{\mathrm{down}} & \mathrm{if}\;F_{\max}^{\left(k\right)}<F_{\mathrm{safe}},\\ 1 & \mathrm{otherwise}, \end{array}\right.$$

where $\gamma_{\mathrm{up}}=1.1$ (larger patches), $\gamma_{\mathrm{down}}=0.9$ (smaller patches), $F_{\mathrm{target}}=0.9$ (conservative threshold), and $F_{\mathrm{safe}}=0.7$ (safe margin). Initial value: $\beta^{\left(0\right)}=0.6$ (top 40% of stressed nodes).

It is noted that the statistical threshold used to define the SRR extent also implicitly accounts for stress concentrations arising at patch boundaries. In the present implementation, the percentile level is selected slightly below the value that would isolate only the peak stress core, based on empirical observations. This results in SRRs that intentionally extend beyond the maximum stress locus, providing a buffer region around the patch perimeter. Such enlargement mitigates sharp stress gradients at patch edges and reduces the risk of localised stress amplification that could otherwise promote interlaminar damage or delamination. This strategy allows for boundary effects to be handled in a stress-driven manner, rather than through purely geometric offsets.

#### 2.1.4. Spatial Clustering via DBSCAN

Let $x_{i}\in R^{2}$ denote the in-plane coordinates of node *i*. The DBSCAN (Density-Based Spatial Clustering of Applications with Noise) algorithm partitions the filtered node set $H^{\left(j^{*}\right)}$ into spatially coherent clusters as follows:

(7) $$\mathrm{DBSCAN}\left(\{x_{i}:i\in H^{\left(j^{*}\right)}\},\varepsilon ,m\right)\;⟶\;\left\{C_{1},\ldots ,C_{K},N_{\mathrm{noise}}\right\},$$

where each $C_{k}\subseteq H^{\left(j^{*}\right)}$ is a cluster of high-failure nodes and $N_{\mathrm{noise}}$ contains isolated outliers corresponding to artificial stress concentrators (e.g., from constraint simplifications).

##### Automatic Parameter Selection

The DBSCAN parameters are determined from mesh characteristics as follows:

(8) $$\begin{array}{cc} \varepsilon & =\alpha_{\varepsilon }\cdot {\bar{h}}_{\mathrm{mesh}}, \end{array}$$

(9) $$\begin{array}{cc} m & =\lceil \beta_{m}\cdot \rho_{\mathrm{local}}\rceil , \end{array}$$

where ${\bar{h}}_{\mathrm{mesh}}$ is the average element size in layer $j^{*}$, $\rho_{\mathrm{local}}$ is the local node density, and $\alpha_{\varepsilon }=2.5$, $\beta_{m}=5$ are calibration constants determined empirically.

#### 2.1.5. Patch Boundary Extraction

For each cluster $C_{k}$, we compute a boundary polygon $P_{k}$ that encloses the high-stress region. Several approaches were evaluated as follows:

(i) Convex hull:
  Fast (O(nlogn)) but cannot capture concave stress concentrations.
(ii) Exporting perimeters from DBSCAN boundary points:
  Extremely fast (none mathematical operations proceeded), but does not work reliably.
(iii) α-shapes:
  Accurate concave hull reconstruction but computationally expensive ($O(n^{2})$) for large clusters.
(iv) Custom boundary algorithm:
  Simple algorithm developed specifically for this application, balancing accuracy and computational efficiency. The algorithm operates in several steps:

Step 1: Geometric Center Calculation

(10) $$c=\frac{1}{|C_{k}|}\sum_{x_{i}\in C_{k}}x_{i}$$

where c represents the geometric centroid of cluster $C_{k}$.

Step 2: Bounding Circle Definition

(11) $$R=\alpha \cdot \max_{x_{i}\in C_{k}}∥x_{i}-c∥,\;\alpha =1.25$$

The scaling factor α ensures complete enclosure of all cluster points.

Step 3: Radial Sector Generation

(12) $$p_{j}=c+R\cdot \left[\begin{array}{c} \cos\theta_{j}\\ \sin\theta_{j} \end{array}\right],\;\theta_{j}=\frac{2\pi j}{N}$$

for j=0,1,…,N−1, with *N* typically set to 36 sectors for 10° angular resolution.

Step 4: Boundary Vertex Selection

(13) $$v_{j}=\arg\min_{x\in C_{k}\setminus V_{j-1}}{∥x-p_{j}∥}_{2}$$

where $V_{j-1}=\left\{v_{0},v_{1},\ldots ,v_{j-1}\right\}$ prevents vertex duplication.

The selective reinforcement region is then defined as

(14) $$\Omega_{k}^{\mathrm{SRR}}=\left\{x\in R^{2}:x\;\mathrm{lies}\;\mathrm{inside}\;\mathrm{polygon}\;P_{k}\right\}$$

where $P_{k}=\left\{v_{0},v_{1},\ldots ,v_{N-1}\right\}$ forms the ordered boundary polygon.

Time complexity $O(N\cdot |C_{k}|)$, with empirical execution is faster than α-shapes. Accuracy: 80–85% relative to α-shape benchmarks, suitable for iterative optimization frameworks where computational efficiency is prioritized.

#### 2.1.6. SRR Material and Orientation Assignment

Each SRR inherits the material and fiber orientation of the removed layer as follows:

(15) $$\Omega_{k}^{\mathrm{SRR}}\leftarrow \left(M_{j^{*}},\theta_{j^{*}}\right).$$

This ensures mass balance: the total material volume remains constant when a full layer is replaced by localized SRRs. Future work will explore optimal material and orientation selection for each patch.

#### 2.1.7. Iterative Laminate Update

Let $L^{\left(k\right)}=\left\{1,2,\ldots ,L_{k}\right\}$ denote the set of layers in the base laminate at iteration *k*, and $S^{\left(k\right)}$ the set of SRRs inserted in previous iterations.

After FEA, the global maximum Tsai–Wu failure index is

(16) $$F_{\max}^{\left(k\right)}=\max_{j\in L^{\left(k\right)}}\;\max_{i\in N}F_{i}^{\left(j\right)}.$$

The algorithm updates the configuration based on structural performance as follows:

Case 1: Lightweighting Mode ($F_{\max}^{\left(k\right)}<1$).

The structure is safe. Remove layer $j^{*}$ and replace with SRRs as follows:

(17) $$\begin{array}{cc} L^{\left(k+1\right)} & =L^{\left(k\right)}\setminus \left\{j^{*}\right\}, \end{array}$$

(18) $$\begin{array}{cc} S^{\left(k+1\right)} & =S^{\left(k\right)}\cup \left\{\Omega_{1}^{\mathrm{SRR}},\ldots ,\Omega_{K}^{\mathrm{SRR}}\right\}. \end{array}$$

Case 2: Strengthening Mode ($F_{\max}^{\left(k\right)}\geq 1$).

The structure is failing. Add SRRs without removing base layers:

(19) $$\begin{array}{cc} L^{\left(k+1\right)} & =L^{\left(k\right)}, \end{array}$$

(20) $$\begin{array}{cc} S^{\left(k+1\right)} & =S^{\left(k\right)}\cup \left\{\Omega_{1}^{\mathrm{SRR}},\ldots ,\Omega_{K}^{\mathrm{SRR}}\right\}. \end{array}$$

Let $W^{\left(k\right)}$ denote the total laminate mass as follows:

(21) $$W^{\left(k\right)}=\sum_{j\in L^{\left(k\right)}}\rho_{j}A_{j}t_{j}+\sum_{\Omega \in S^{\left(k\right)}}\rho_{\Omega }\left|\Omega \right|t_{\Omega },$$

where $\rho_{j}$ is material density, $A_{j}$ is layer area, $t_{j}$ is ply thickness, and |Ω| is SRR area.

#### 2.1.8. Convergence Criteria

The optimization terminates when any of the following conditions are met:

(a) Performance-based:
  (22) $$F_{\max}^{\left(k\right)}<1\;\mathrm{and}\;\left|\frac{W^{\left(k+1\right)}-W^{\left(k\right)}}{W^{\left(k\right)}}\right|<\epsilon_{W},$$
  where $\epsilon_{W}=0.01$ (1% weight change threshold).
(b) Variability-based:
  (23) $$\max_{j\in L^{\left(k\right)}}{\mathrm{CV}}_{j}\;<\;{\mathrm{CV}}_{\min},$$
  where ${\mathrm{CV}}_{\min}=0.3$ indicates all remaining layers have nearly uniform stress distributions.
(c) Structural minimum:
  (24) $$|L^{\left(k\right)}|\leq L_{\min},$$
  where $L_{\min}=2$ prevents complete removal of the base laminate.
(d) Maximum iterations:
  (25) $$k>k_{\max},$$
  where $k_{\max}=200$ prevents excessive computation.

The algorithm prioritizes criteria (a) and (b) as indicators of successful convergence, while (c) and (d) serve as safety limits.

### 2.2. Algorithm Implementation

**Figure 1:**
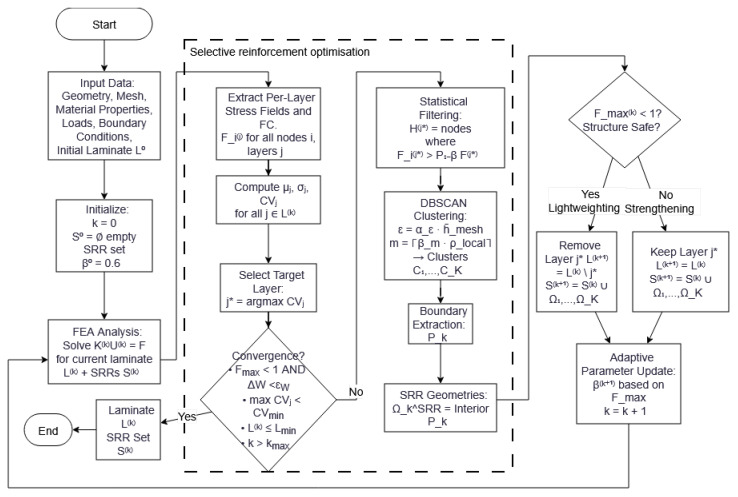
Overview of the Selective Reinforcement Optimization workflow.

The Selective Reinforcement Optimization (SRO) workflow is implemented as a fully automated pipeline operating on layer-wise FEA results. Although any finite element package can be used, the present work employs the Abaqus(version 6.14 or higher)/Simulia environment from Dassault Systèmes because it exposes a native Python scripting interface, enabling seamless execution of the entire SRO algorithm within the solver environment. All post-processing, clustering, perimeter extraction, and geometry reconstruction procedures are written in Python (version 3 or higher) and executed automatically after each FEA iteration. The overall workflow is summarized in Figure 1.

#### 2.2.1. Extraction of Layer-Wise Stress Data

**Figure 2:**
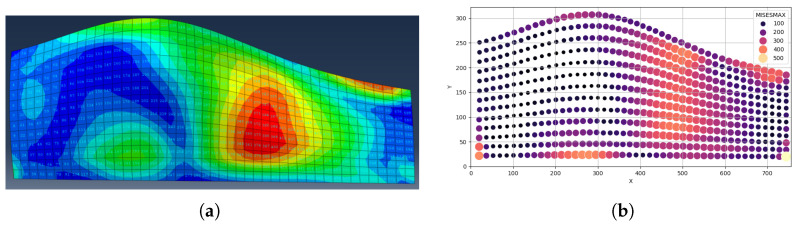
(**a**) FEA stress distribution visualization for a single laminate ply. (**b**) Draw exported nodal stress data used for SRO preprocessing.

For each iteration, Abaqus computes the stress field for each laminate ply. Nodal coordinates, ply indices, and scalar stress measures (e.g., von Mises or Tsai–Wu failure index) are exported to CSV files for downstream processing. Figure 2a shows the FEA stress distribution in the Abaqus interface, while Figure 2b shows the corresponding raw exported nodal dataset used as input for the threshold clustering.

To select the target ply for SRR construction, the algorithm computes the coefficient of variation (CV) of the chosen failure metric for each ply and selects $j^{*}=\arg\max_{j}$, ${\mathrm{CV}}_{j}$. A high CV signals that stresses are concentrated in localized regions of the ply (rather than uniformly distributed), making that ply a suitable candidate for selective reinforcement. The full mathematical formulation of this criterion is provided in Section 2.1.1 (Layer-wise Statistical Characterisation).

#### 2.2.2. Threshold Filtering of High-Stress Nodes

**Figure 3:**
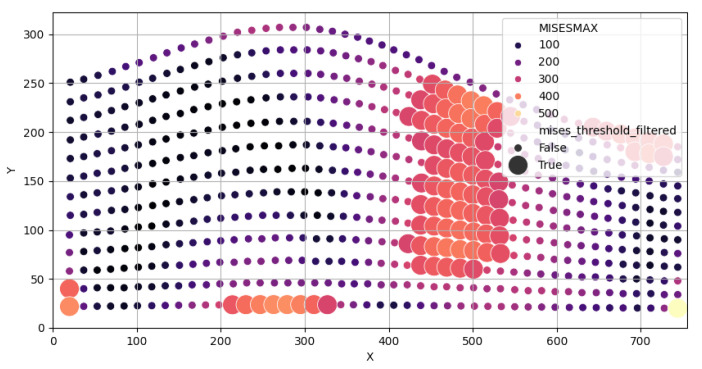
Stress field after percentile-based threshold filtering.

Threshold is applied according to the statistical formulation presented in Section 2.1.2 (Statistical node filtering of the ply’s failure index distribution). Nodes exceeding the adaptive percentile threshold form the set of statistically significant stress locations. Figure 3 illustrates the resulting filtered stress field.

#### 2.2.3. DBSCAN Clustering of Significant Stress Regions

**Figure 4:**
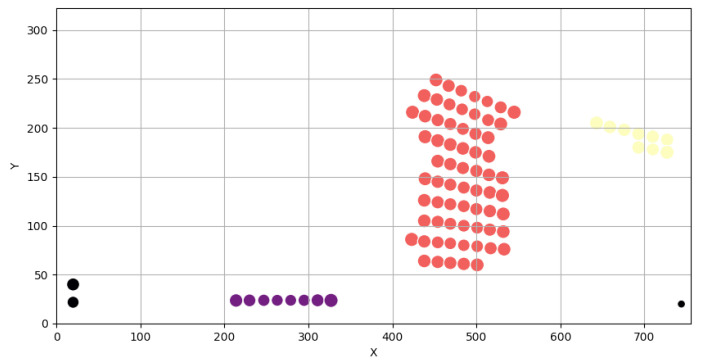
High-stress regions identified using DBSCAN clustering.

The filtered nodes are spatially grouped using DBSCAN, which automatically identifies coherent stress clusters while eliminating isolated points caused by local mesh artifacts or artificially stiff BC definitions. Cluster parameters (radius and minimum density) are derived from the mean nodal spacing of the FE mesh. Figure 4 shows the resulting clustered stress regions, with different colors representing individual clusters and black points indicating outliers.

DBSCAN is selected because it does not require the number of clusters to be prescribed a priori and can naturally identify arbitrarily shaped stress regions while rejecting isolated points as noise. The neighborhood radius is defined based on the average inter-nodal spacing of the FE mesh, providing a physically meaningful spatial scale for grouping stress nodes into coherent regions associated with potential reinforcement patches.

#### 2.2.4. Perimeter Extraction via Custom Concave-Hull Approximation

Once stress clusters are identified, SRO must construct a manufacturable boundary around each reinforcement region. Several geometric extraction methods can be used as follows:

1. α-shape algorithm(primary method):
  α-shapes offer the most accurate concave hull and are therefore the default perimeter extraction method in SRO.
2. Convex hull (optional):
  Very efficient but ignores concavities, overestimates patch size, and wastes material.
3. DBSCAN border points (optional):
  Extremely fast (virtually immediate, since no mathematical operations were made) because it uses DBSCAN’s native border labeling, but it is unreliable in practice—often failing to detect entire sections of the boundary.
4. Custom fast perimeter mapping algorithm (proposed):
  A robust compromise between geometric accuracy and computational efficiency. It avoids the cost of α-shapes while producing sufficiently precise boundaries for composite manufacturing, where ply cutting tolerance limits the practical need for sub-millimetric accuracy.
  Given that reinforcement layers are placed in an unconsolidated state and cannot be cut with perfect precision, the custom perimeter extraction is generally sufficient and is used in applications requiring hundreds of SRO iterations.

##### Two-Step Custom Perimeter Extraction (Visualized in Figure 5)

**Figure 5:**
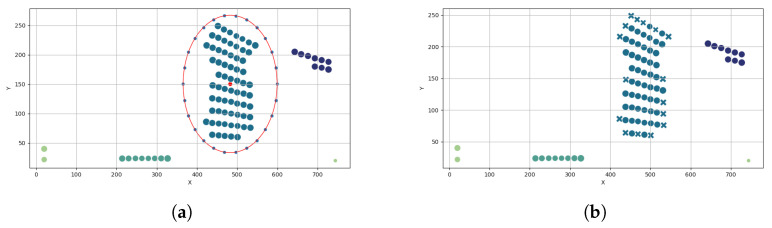
(**a**) Step 1: Radial subdivision of the stress cluster using a bounding circle. (**b**) Step 2: Concave boundary extraction via nearest-point selection per angular sector.

Step 1 Radial subdivision using a bounding circle
  A circle is constructed around the cluster by taking the maximum radial distance from the cluster centroid and scaling it ×1.25. This circle is then discretised into angular sectors (usually 72 segments is enough; possibly more for complex regions with internal cavities). (Figure 5a)
Step 2 Nearest-point assignment per sector For each angular sector, the closest cluster node lying inside the circle is selected as a perimeter vertex. Already-selected nodes are excluded to ensure an ordered, non-overlapping perimeter. (Figure 5b)

The resulting sequence of perimeter points approximates the cluster’s concave hull and is sufficiently accurate for spline reconstruction and subsequent patch generation.

The boundary extraction step converts discrete clustered nodes into a closed, CAD-compatible curve suitable for patch generation. While α-shapes are used as the primary approach due to their ability to capture concave geometries, simpler fast approximations are also considered when computational efficiency is prioritised. This step ensures that SRO outputs are directly manufacturable rather than abstract point sets.

#### 2.2.5. Reconstruction of the Reinforcement Patch

**Figure 6:**
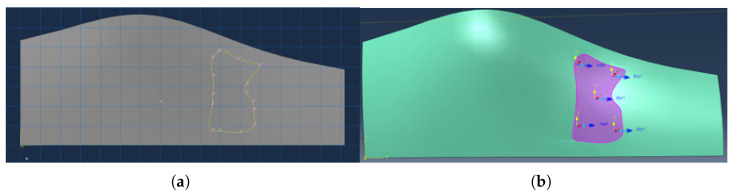
(**a**) Spline-based reconstruction of the reinforcement patch boundary (**b**) SSR material orientation visualization.

The extracted perimeter nodes are transferred back into Abaqus, where a 3d closed spline curve is constructed using positions of perimeter nodes to define the reinforcement patch geometry. The spline conforms to the laminate surface and encloses the SRR derived from clustering. Figure 6a shows a typical SRR outline reconstructed on the finite element mesh. SSR orientation Figure 6b is set as orientation of the mother layer, since no additional optimisation is performed at this point.

#### 2.2.6. Integration of the Patch into the Structural Model

The SRR spline defines a local region where reinforcement is either added or reallocated depending on the structural state:

- Lightweighting mode:
  If all failure indices remain below unity, the selected ply is removed globally and reintroduced as a local SRR patch.
- Strengthening mode:
  If any failure index exceeds unity, the ply is retained and an additional reinforcement patch is added at the SRR location.

**Figure 7:**
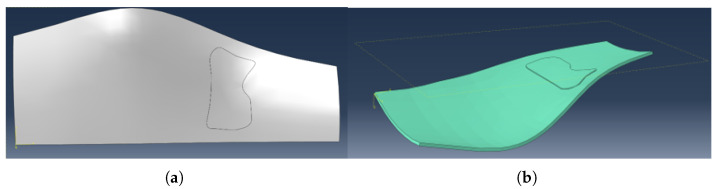
Final 3D visualization of the laminate with integrated reinforcement patch on “part” mode (**a**) and “property” (**b**) mode with thickness rendering.

The resulting geometry is assigned its corresponding material orientation and included in the next FEA iteration. A representative 3D view is shown in Figure 7.

**Figure 8:**
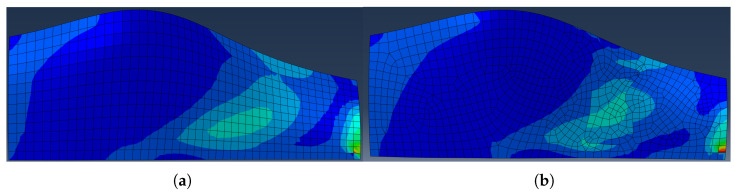
Representative Tsai–Wu failure index distribution in the panel (**a**) before and (**b**) after SRO, illustrating qualitative stress redistribution due to selective reinforcement.

While the previous figures demonstrate how SRO generates manufacturable reinforcement geometries, it is also important to assess how this redistribution affects the internal stress state of the laminate. Therefore, a representative comparison of the Tsai–Wu failure index fields before and after the introduction of selective reinforcement is presented on Figure 8 to provide qualitative insight into the structural response.

As observed in Figure 8, the pre-optimisation laminate exhibits a broadly distributed failure index. Outside the SRR, the laminate shows more uniform and relaxed stress state, indicating that material redistribution effectively channels the load through the intended reinforcement paths.

It is noted that both distributions display a pronounced local stress concentration in the lower-right corner of the panel. This feature is not associated with the structural behaviour of interest but is attributed to the simplified boundary condition representation used in the finite element model, where a minimal set of constrained nodes (three-point supports, with one node fully fixed and others partially constrained) is employed to prevent rigid-body motion while allowing free in-plane deformation. Such idealised constraints are known to introduce artificial stress singularities that are highly mesh- and formulation-dependent. Since this region is remote from the load-carrying paths and does not influence the SRR generation, it is regarded as a numerical artefact and is therefore excluded from the interpretation of the stress redistribution results.

For this reason, the figure is intended solely for qualitative comparison of stress patterns rather than quantitative evaluation of failure levels.

#### 2.2.7. Iterative Loop and Convergence

The iterative optimization loop continues until both conditions are satisfied as follows:

- Performance stability:
  No failure criterion exceeds unity, and structural performance change between iterations is negligible.
- Variance stability:
  The highest coefficient of variation across all layers falls below the variance threshold, indicating uniformly distributed stress.

The final output consists of a laminate with redistributed reinforcement mass localized precisely at structurally critical regions.

## 3. Results

### 3.1. Case Study Definition

**Figure 9:**
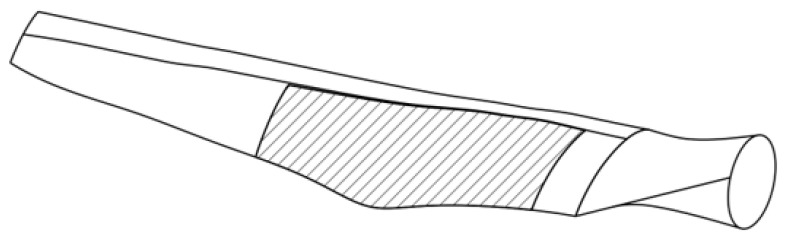
Schematic of the composite panel considered in the case study.

The optimisation study considers a structural panel representative of a skin panel within a utility-scale wind turbine blade. The blade is assembled from multiple composite panels; the specimen used here is one such panel and represents the structural domain to which Selective Reinforcement Optimization (SRO) is applied (schematic in Figure 9). Analyses are performed under the assumptions of thin composite plate theory, i.e., the panel response is dominated by in-plane membrane actions and bending effects that are consistent with classical laminate plate formulations; consequently the loading cases considered here are expressed in terms of in-plane stress resultants and shear resultants. The choice of thin-plate modelling is appropriate because the panel’s laminate thickness is small relative to its in-plane dimensions and because the operational load spectra considered (see below) impose predominantly in-plane forces on the skin panels [2].

The mesh size was selected as 0.03–0.07 times the characteristic dimension (15–25 mm), resulting in approximately 600 nodes total across the laminate. This element size falls within the empirically determined adequate range of 0.01–0.1 for the SRO process, balancing computational cost with sufficient resolution for stress gradient capture. It is noted that the manufacturing process for composite patches inherently imposes geometric tolerances that exceed the precision requirements of sub-millimeter mesh refinement.

The panel comprises a multi-material composite laminate (several ply materials and orientations); therefore, material-specific engineering constants are modelled directly in the finite element model and are not reported as a single global set here. Geometric dimensions of the panel and the three canonical design load cases considered in this study are summarised in Table 1.

The three load cases represent the operational extremes commonly encountered by a blade panel as follows:

- LC1—Stationary (gravity-dominated): nominal aerodynamic loading is absent; the panel is loaded primarily by gravity-induced in-plane resultants and self-weight effects.
- LC2—Wind normal to the blade (suction side loading): aerodynamic pressure produces in-plane tensile/compressive resultants that act approximately normal to the blade chord locally (this produces a characteristic stress band on the skin).
- LC3—Wind tangential to the blade (tangential/torsional loading): aerodynamic loading produces in-plane shear-dominated and tangential normal resultants, approximating torsional demand on the panel.

SRO was executed four times for the panel: once for each individual load case (LC1–LC3) and once for a combined design run that enforces reinforcement to satisfy all three load cases simultaneously. Finite element analyses were carried out for each load condition independently; the separate single-load runs allow SRO to tailor reinforcement geometry specifically to each loading direction, while the combined run evaluates the feasibility of a unified reinforcement strategy that meets all cases.

### 3.2. SRO Optimisation Results for Individual and Multi-Load Conditions

The Selective Reinforcement Optimisation (SRO) procedure was applied to the composite blade panel described in Section 3.1 under four optimisation scenarios as follows:

(a) Optimisation with respect to LC1 only,
(b) Optimisation with respect to LC2 only;
(c) Optimisation with respect to LC3 only;
(d) Optimisation with respect to the combined action of LC1 + LC2 + LC3.

Each optimisation run was performed independently. For the three single-load cases, the SRO algorithm used only the Tsai–Wu index field generated by the respective load condition to determine the target layer, identify statistically significant stress concentrations, and construct the Selective Reinforcement Regions (SRRs).

The multi-load optimisation was performed differently: all three load cases were evaluated at every iteration, and SRO acted on the load case exhibiting the currently highest failure index. This sequential multi-case treatment avoids the limitations of classical envelope methods, which can be overly conservative for anisotropic laminates. Because reinforcement added to satisfy one directional loading may beneficially alter the response under another, evaluating load cases separately and iteratively allows SRO to exploit anisotropic interactions that envelope analysis cannot capture.

Table 2 summarises the quantitative results obtained in each optimisation scenario. For each case, the following values are reported:

- The initial maximum Tsai–Wu index, $F_{\max}^{\left(0\right)}$;
- The final maximum Tsai–Wu index at convergence, $F_{\max}^{\left(\mathrm{opt}\right)}$;
- The panel mass before and after optimisation;
- The number of SRO iterations required to reach convergence.

The results confirm that SRO consistently identifies stress-driven reinforcement regions and achieves weight reduction while maintaining structural safety across all load conditions. As expected, the multi-load optimisation exhibits a smaller mass reduction compared to the single-load cases, reflecting the increased demands imposed by simultaneously satisfying three distinct loading scenarios.

### 3.3. Effect of Cluster-Selection Strategy on SRO Convergence

The Selective Reinforcement Optimisation algorithm requires choosing one stress cluster per iteration on which the SRR is constructed. To quantify how this choice influences the optimisation trajectory, five selection strategies were examined:

(i) **Largest cluster**—the cluster containing the greatest number of filtered nodes.
(ii) **Peak-stress cluster**—the cluster containing the global maximum of the Tsai–Wu index.
(iii) **Mean-stress cluster**—the cluster with the highest average Tsai–Wu index.
(iv) **Cumulative-stress cluster**—the cluster with the highest sum of Tsai–Wu indices.
(v) **Mega-SRR (union)**—a single SRR enclosing all clusters simultaneously.

**Figure 10:**
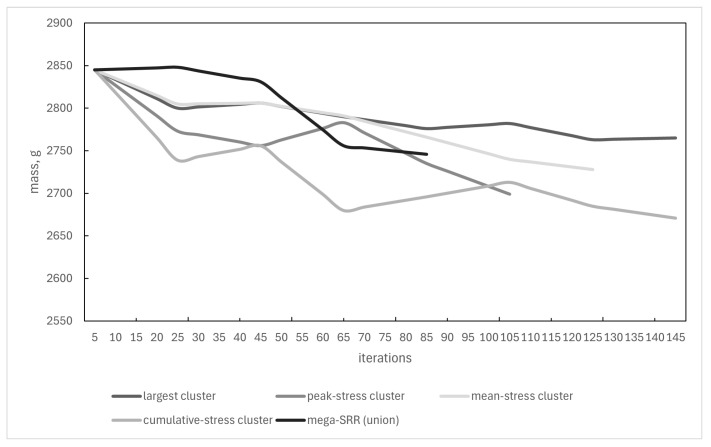
Evolution of panel mass for five cluster-selection strategies.

Each strategy was applied to the same baseline laminate and the same load case, with identical filtering and DBSCAN parameters. Figure 10 shows the evolution of panel mass as a function of iteration number. All strategies exhibit a monotonic decrease in mass, but the rate and final value differ. The “cumulative-stress” and “peak-stress” strategies produce the most aggressive lightweighting, reaching the lowest final mass. The “largest-cluster” strategy reduces mass more slowly but maintains a smoother trajectory, reflecting its more conservative redistribution pattern. The “mega-SSR” and “mean-stress” criteria fall between these extremes.

**Figure 11:**
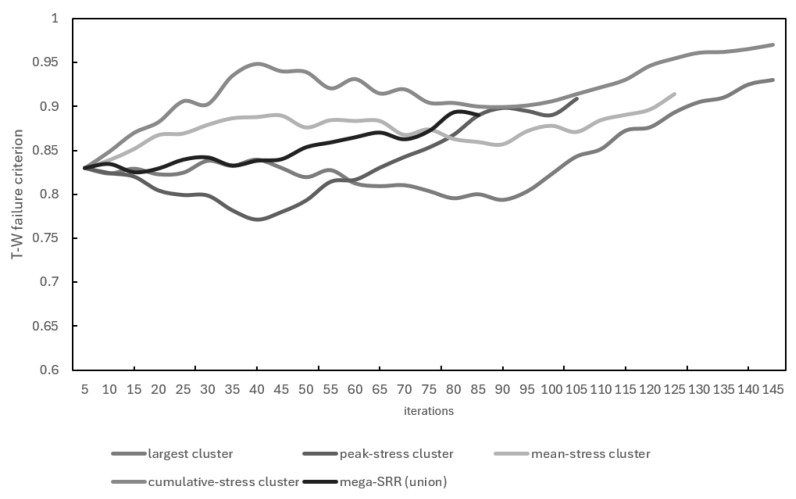
Evolution of $F_{\max}$ for five cluster-selection strategies.

Figure 11 reports the evolution of the maximum Tsai–Wu index times safety coefficient 1.15 $F_{\max}\cdot k_{safety}$. All strategies remain within the safe regime $F_{\max}\cdot k_{safety}<1$ throughout the run, though with different stability characteristics.

The “largest-cluster” option provides the most stable $F_{\max}$ behaviour, with minimal oscillation. The cumulative-stress strategy produces the lowest mass but also the largest fluctuations in $F_{\max}$, indicating a more aggressive and less stable reinforcement pattern. The peak-stress and mean-stress strategies again occupy an intermediate region, showing modest oscillations with no crossing of the failure threshold.

## 4. Discussion

The Selective Reinforcement Optimization (SRO) method developed in this work has demonstrated its potential for substantial weight reductions in laminated composite panels subjected to in-plane loads. By iteratively removing full layers and replacing them with localized reinforcement patches only where needed, the method enables non-uniform material redistribution that aligns closely with critical stress paths [19].

The results show that the method achieves the most significant weight reductions under relatively simple load conditions where stress concentrations are sharply defined and shared across multiple plies. In these scenarios, a single Stress-Driven Reinforcement Region (SRR) can effectively reinforce multiple layers, leading to minimal material use and maximum efficiency. This is in contrast to classical topology optimization techniques, which tend to produce density-based solutions requiring extensive post-processing to yield discrete patch geometries.

When SRO is applied to more complex design cases—such as the multi-load condition where all three load cases are active sequentially—its efficiency diminishes. This is because critical stress zones often do not overlap across layers or between load cases, requiring more SRRs to maintain safety. Under such multi-axial or conflicting loading, the structural demands vary by orientation, weakening the alignment of the reinforcement geometry across plies. In extreme cases, the algorithm is forced to retain many full layers, resulting in smaller net weight reductions.

The present study focuses on in-plane loading conditions, consistent with the classical thin composite plate theory. This framework is well suited for membrane-dominated structures. Nevertheless, the SRO algorithm itself is not inherently restricted to in-plane loads: it operates on general stress fields extracted from FEA. In principle, out-of-plane bending, transverse shear, or even dynamic loading cases could be incorporated, provided that the corresponding layer-wise stress measures are available. Extending SRO to such loading regimes, and assessing its performance under plate bending and transient responses, constitutes an important direction for future work.

Unlike conventional topology optimization methods that generate continuous density fields requiring interpretation for composite manufacturing, SRO produces discrete, manufacturable reinforcement geometries directly. While TO theoretically achieves greater mass reduction (in benchmark studies it is claimed up to 30%), it often yields designs incompatible with layer-based composite fabrication. In contrast, SRO achieves 10–20% mass reduction while maintaining 100% manufacturability, requiring no post-processing interpretation. Compared to classical size/shape optimization for composites, which adjusts global parameters (ply thickness, ply angles), SRO’s localized material redistribution approach more effectively addresses stress concentrations without altering the overall laminate architecture.

### Limitations and Future Work

Several implementation challenges remain. The current clustering approach relies on DBSCAN with a neighborhood radius derived from the average inter-nodal spacing [20], assuming uniform mesh density. In practice, however, adaptive meshing is common in FE simulations, especially near geometric singularities or areas of a high stress gradient. Under such conditions, stress nodes may be over- or under-clustered depending on local density. A more adaptive definition of the DBSCAN epsilon parameter would improve generality.

When the majority of a ply is highly stressed, the stress filtering process may select almost all nodes. In these cases, SRO behaves more like a layer-wise topology optimization strategy and loses its patch selectivity. This defeats the purpose of SRR localization and introduces unnecessary computational effort and manufacturing complexity.

When the majority of a ply is highly stressed, the stress filtering process may select almost all nodes. In these cases, SRO behaves more like a layer-wise topology optimization strategy and loses its patch selectivity. Similar conditions are observed when clusters are geometrically irregular with near-global stress coverage (typically >70% of ply area) and concave features, where the radial sweep may under-approximate boundaries and therefore reduce SRO to typical topology optimization. Conversely, excessive cluster fragmentation can occur, where numerous small SRRs slow convergence without affecting structural validity. However, these pathological cases occurred rarely and primarily under extreme multi-axial loading where stress patterns exhibit no clear localization.

Stress concentrations at patch boundaries are implicitly mitigated through the adaptive statistical threshold used to define SRRs, which is intentionally set slightly conservative based on empirical tuning. However, no quantitative assessment of interlaminar stress reduction or delamination risk is provided in this study. A systematic evaluation of patch-edge stress gradients using interlaminar stress measures or cohesive criteria is identified as an important direction for future work.

These limitations suggest several directions for future research. Adaptive clustering schemes that account for local mesh density should be explored. Patch generation strategies that exploit cross-layer stress coherence could enhance performance for multi-load cases. In addition, while Tsai–Wu indices were used here due to their direct availability from Abaqus, future implementations may operate directly on stress components and alternative failure criteria, broadening the applicability of SRO to different composite systems and design frameworks.

Additionally, the current implementation assigns each SRR the same material and fiber orientation as its parent layer. While this ensures mass balance and manufacturing simplicity, optimizing patch-specific fiber orientations could yield further structural improvements and represents a promising direction for future research.

## 5. Conclusions

This study introduced Selective Reinforcement Optimization (SRO), a stress-driven framework for generating localized reinforcement patches in laminated composites. The main conclusions are as follows:

- SRO achieves weight reductions on the order of 10–20% while maintaining failure indices below unity for all investigated load cases.
- The method is inherently compatible with layer-based composite manufacturing, producing discrete reinforcement geometries without density interpretation or post-processing.
- SRO is most efficient when stress localization is coherent across plies, whereas performance decreases for highly heterogeneous multi-load conditions.
- The framework integrates FEA automation, statistical stress filtering, clustering, and geometric boundary extraction into a unified design loop.

Overall, SRO provides a practical pathway toward stress-informed, manufacturable optimization of composite laminates and offers a complementary alternative to conventional topology optimization for additive and automated composite fabrication.

## Figures and Tables

**Table 1:** Load cases and dimensions of the composite structure.

Parameter	LC1	LC2	LC3
$N_{x}$ (kN)	180	250	−300
$N_{y}$ (kN)	300	220	−140
$Q_{xy}$ (kN)	600	−120	350
Length (mm)	720		
Width (mm)	380		
*p* (kPa)	1.53		
Baseline weight (g)	2845.659		

Note. $N_{x},N_{y},Q_{xy}$ denote the in-plane resultants in the local *x* and *y* directions and the in-plane shear resultant, respectively (units reported in the table).

**Table 2:** Summary of Selective Reinforcement Optimisation outcomes for single-load and multi-load scenarios.

Metric	LC1	LC2	LC3	Multi-Load
Initial $F_{\max}^{\left(0\right)}$	0.84	0.84	0.84	0.84
Final $F_{\max}^{\left(\mathrm{opt}\right)}$	0.96	0.88	0.89	0.94
Initial mass (g)	2845.659	2845.659	2845.659	2845.659
Final mass (g)	2583.28	2610.94	2622.58	2731.52
Iterations to convergence	62	87	34	139

## Data Availability

The original contributions presented in this study are included in the article. Further inquiries can be directed to the corresponding author.

## Abbreviations

The following abbreviations are used in this manuscript:

SRO	Selective Reinforcement Optimisation
SRR	Selective Reinforcement Region
DBSCAN	Density-based scan
ML	Machine Learning (algorithm)
FEA	Finite Element Analysis
